# Advancing yeast metabolism for a sustainable single carbon bioeconomy

**DOI:** 10.1093/femsyr/foaf020

**Published:** 2025-04-17

**Authors:** Miriam Kuzman, Özge Ata, Diethard Mattanovich

**Affiliations:** Institute of Microbiology and Microbial Biotechnology, Department of Biotechnology and Food Science, BOKU University, 1190, Vienna, Austria; Austrian Centre of Industrial Biotechnology (ACIB), 1190, Vienna, Austria; Institute of Microbiology and Microbial Biotechnology, Department of Biotechnology and Food Science, BOKU University, 1190, Vienna, Austria; Austrian Centre of Industrial Biotechnology (ACIB), 1190, Vienna, Austria; Institute of Microbiology and Microbial Biotechnology, Department of Biotechnology and Food Science, BOKU University, 1190, Vienna, Austria; Austrian Centre of Industrial Biotechnology (ACIB), 1190, Vienna, Austria

**Keywords:** methanol, formate, carbon dioxide, bioeconomy, sustainability

## Abstract

Single carbon (C1) molecules are considered as valuable substrates for biotechnology, as they serve as intermediates of carbon dioxide recycling, and enable bio-based production of a plethora of substances of our daily use without relying on agricultural plant production. Yeasts are valuable chassis organisms for biotech production, and they are able to use C1 substrates either natively or as synthetic engineered strains. This minireview highlights native yeast pathways for methanol and formate assimilation, their engineering, and the realization of heterologous C1 pathways including CO_2_, in different yeast species. Key features determining the choice among C1 substrates are discussed, including their chemical nature and specifics of their assimilation, their availability, purity, and concentration as raw materials, as well as features of the products to be made from them.

## Teaser

The recent advancements in engineering of single carbon (C1) utilization pathways in yeast biotechnology for future C1-based processes are discussed.

## Introduction

**Figure 1.**
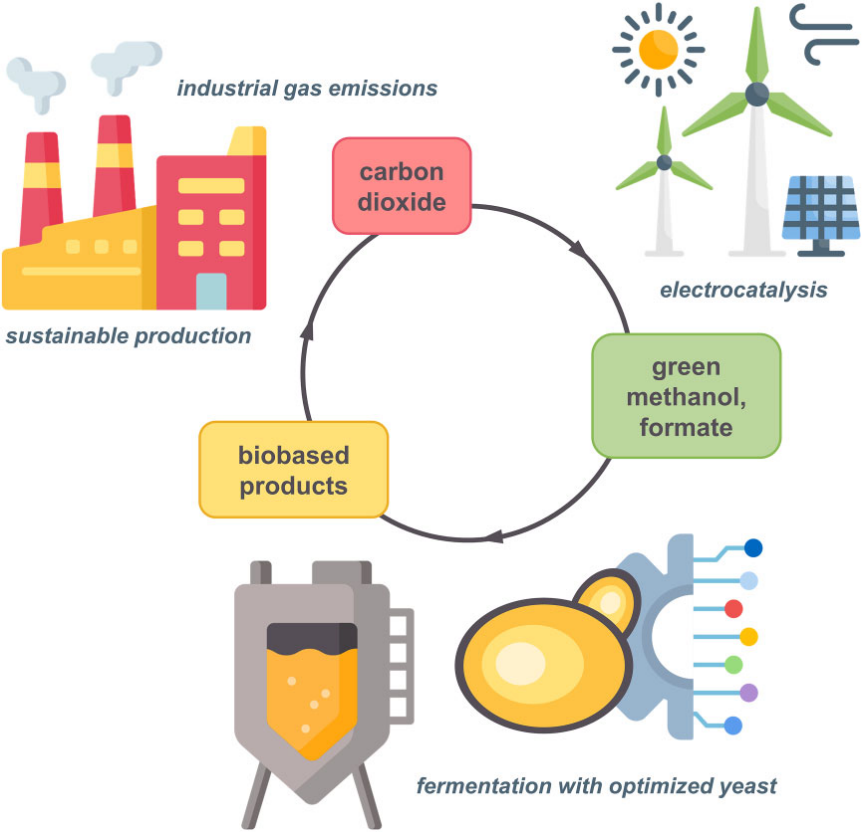
Schematic overview of a circular single carbon bioeconomy. CO_2_ emissions from various industries can be harvested and reduced to methanol and formate, and serve as carbon and energy sources for the conversion to value-added products by C1-assimilating yeast strains.

The utilization of single carbon (C1) substrates, such as methanol, CO₂, and formate, has gathered significant interest in the field of yeast biotechnology due to their potential to contribute to sustainable and renewable biotechnological processes (Fabarius et al. 2021). These C1 compounds can be harnessed as alternative, non-food carbon sources, offering opportunities to mitigate environmental issues such as CO₂ emissions and to reduce dependence on fossil-based raw materials (Fig. 1).

While naturally occurring methylotrophic yeasts have already been exploited for their ability to assimilate methanol (Sreekrishna and Kropp 1996, Gellissen 2000), ongoing research seeks to improve the efficiency of these processes through metabolic engineering and synthetic biology approaches, to expand the host and the substrate spectrum.

Nature has evolved several single-carbon assimilation pathways, some of which overlap (Baumschabl et al. 2024). The ribulose monophosphate (RuMP) and xylulose monophosphate (XuMP) cycles, both involved in methanol assimilation, share similarities with the Calvin–Benson–Bassham (CBB) cycle, which is used for CO₂ fixation. When formaldehyde is further oxidized to formate, it can be assimilated via the serine cycle or the linear reductive glycine (rGly) pathway. Another linear pathway for CO_2_ assimilation is the Wood Ljungdahl pathway. Only the XuMP cycle for methanol assimilation is natively found in yeasts (Rußmayer et al. 2015), as for example in *Komagataella phaffii* (formerly known as *Pichia pastoris*) and *Ogataea polymorpha* (previously named *Hansenula polymorpha*).

Historically, methanol was the first C1 source used in biotechnology, primarily for the production of single-cell protein (SCP) using natural consumers such as *K. phaffii*. With the emerging concept of a methanol-based bioeconomy, sustainable production processes for green methanol were explored. As genetic engineering tools advanced, CO₂ emerged as a potential carbon source, enabling the development of engineered strains capable of assimilating it. More recently, formate has gained attention as a promising carbon source for microbial processes, offering the advantages of being liquid and non-toxic, unlike CO₂ and methanol.

In this minireview, we aim to discuss the significance of the three C1 substrates in yeast biotechnology, highlighting the latest research on various yeast species and the metabolic engineering efforts to introduce and improve C1 assimilation pathways, with the potential to be utilized in C1-based bioeconomies (Table 1).

**Table 1.** Overview of engineered yeast species for the utilization of single carbon substrates.^a^

C1 source	Yeast species	Metabolic engineering	Growth	Reference
Methanol	*Komagataella phaffii*	Starting point: ∆*FLD* strain for less carbon loss. Problem: disbalance in NAD⁺/NADH ratio. Introduction of dual-enzyme complex (Aox1–Das1) to mitigate formaldehyde accumulation; overexpression of isocitrate dehydrogenase to balance NAD⁺/NADH ratio. Heterologous expression of *MOX* from *Ogataea polymorpha* to improve methanol transformation rate.	Strain DF02-1: OD_600_ 4.28 times higher than ∆*FLD* strain under 1% methanol Strain DF02-4: OD_600_ 4.08 times higher than ∆*FLD* strain under 3% methanol	Wang et al. (2024) and Wang et al. (2022)
	*Saccharomyces cerevisiae*	Introduction of *Komagataella phaffii* XuMP cycle modules to convert methanol directly into pyruvate; the yeast consumed 1.04 g/l of methanol.	Strain TACDS2: OD_600_ increase of 3.13%	Dai et al. (2017)
		Introduction of (i) heterologous XuMP cycle, (ii) hybrid XuMP cycle with bacterial Mdh replacing AOX1, and (iii) heterologous RuMP cycle; RuMP cycle found to be most effective in methanol assimilation. Overexpression of *SFA1* to enhance formaldehyde dissimilation and energy production; demonstrated inherent methanol assimilation capacity improved through adaptive laboratory evolution.	No growth on methanol as a sole carbon source (co-substrate: yeast extract)	Espinosa et al. (2020)
		Modular circuit strategy to engineer synthetic methylotrophic strain, resulting in enhanced methanol utilization and the production of value-added bioproducts such as flaviolin.	Strain CX01F: maxOD = 2.0 µ_max = 0.051 h^−1^	Zhan et al. (2023)
		SCRaMbLE genome recombination technology for genome rearrangement; evolved strain metabolizes methanol via Adh2-Sfa1-rGly (ASrG) pathway.	Strain SCSA001: maxOD = 0.547 µ_max = 0.0153 h^−1^	Guo et al. (2024a)
	*Yarrowia lipolytica*	Chimeric MUT pathway integrating elements from RuMP and XuMP cycles, enabling the yeast to assimilate methanol as a sole carbon source and achieving a methanol assimilation level of 1.1 g/l per 72 h.	No growth on methanol as sole carbon source (co-substrate: glucose)	Wang et al. (2021)
		Heterologous XuMP cycle from *K. phaffii* introduced alongside a xylulose utilization pathway; optimized through peroxisomal compartmentalization.	No growth on methanol as a sole carbon source. Strain Yl-004: with xylose as co-substrate: maxOD = 9.0	Zhang et al. (2023)
CO_2_	*Komagataella phaffii*	Integration of the CBB cycle for CO₂ fixation using methanol as an energy source; enhanced by adaptive laboratory evolution to improve ATP availability and balance pathway fluxes with lower activities of the mutated enzymes Nma1 and Prk.	µ_max = 0.018 h^−1^ (for the evolved strain)	Gassler et al. (2020) and Gassler et al. (2022)
	*Komagataella phaffii*	Production of lactic and itaconic acid from CO_2_ via the synthetic CBB cycle.	µ_max = 0.007–0.009 h^−1^	Baumschabl et al. (2022)
	*Saccharomyces cerevisiae*	Expression of RuBisCO and PRK to restore redox balance during ethanol fermentation; reduced glycerol by-product formation, increasing ethanol yield.	IMU033 strain: ca. µ_max = 0.09 h^−1^ (co-substrate: galactose)	Guadalupe-Medina et al. (2013)
		Co-expression of the RuBisCO-PRK module to improve xylose fermentation and ethanol production via redox balance restoration.	Strain SR8c + prk strain: ca. maxDCW: 1.1 g/l (co-substrate xylose) YSC000/110/111 strains: ca. maxOD = 5 (co-substrate glucose in YP medium) YSX4C222 strain: ca. maxOD = 6 (co-substrate xylose-maltose in YP medium)	Xia et al. (2017) and Li et al. (2017)
		Overexpression of pyruvate carboxylase (PC) to increase CO₂ fixation via anaplerotic pathways for the production of TCA intermediates (malate, fumarate, and succinate) and amino acids.	RWB525 strain: ca. µ_max = 0.1 h^−1^ (co-substrate glucose) CTMAE strain: ca. maxOD = 70 (co-substrate xylose) Engineered FMME-002 strain: ca. maxOD = 5 (co-substrate glucose)	Zelle et al. (2008), Xu et al. (2012), and Kang et al. (2022)
	*Kluyveromyces marxianus*	Co-expression of Type I and II RuBisCOs to enhance ethanol production through improved CO₂ fixation and redox balance.	ca. maxOD = 8 (co-substrate glucose)	Ha-Tran et al. (2021)
Formate	*Komagataella phaffii*	Identification of a native oxygen-tolerant reductive glycine (rGly) pathway, enabling slow growth on methanol, formate, and CO₂ without heterologous gene expression.	µ_max = 0.002 h^−1^	Mitic et al. (2023)
	*Saccharomyces cerevisiae*	Demonstrated net glycine production through the endogenous rGly pathway; key metabolite balancing (C1-THF) and reducing power generation identified as bottlenecks.	Evolved clones from the VBS10 strain: ca. µ_max = 0.1 h^−1^ (co-substrate glucose)	Gonzalez De La Cruz et al. (2019) and Bysani et al. (2024)
	*Komagataella phaffii* and *Saccharomyces cerevisiae*	Engineered with a synthetic C1-assimilation pathway (MFORG), allowing mixotrophic utilization of methanol/formate with CO₂ fixation via rGly; *K. phaffii* exhibited superior performance, proof-of-concept production of lactic and 5-aminolevulinic acid (ALA) via MFORG pathway was shown.	*K. phaffii* PMORG09 strain: µ_max = 0.019 h^−1^ *S. cerevisiae* SMFORG01 strain: *ca*. µ_max = 0.006 h^−1^	Guo et al. (2024b)
	*Yarrowia lipolytica*	A native glycine synthesis pathway, supported by a glyoxylate and threonine-based serine pathway to promote formate assimilation via glycine, adaptive laboratory evolution improved formate tolerance and growth on formate.	Evolved strain (M25-70) exhibited a 39% increase in OD600. ca. maxOD = 2.5	Chen et al. (2024)

a In instances where the original paper did not provide specified values, the approximate specific growth rates are given as extracted from the relevant plots for comparison. When there is a co-substrate used in addition to the C1 sources, this is indicated in the *Growth* column.

## Methanol

Methanol is a one-carbon source that can be produced renewably through the hydrogenation or electrochemical reduction of CO₂ (Borisut and Nuchitprasittichai 2019), often referred to as green methanol. Its feasibility for biotechnological processes is supported by several advantages. First, its liquid state at room temperature facilitates easy transport and storage, and its miscibility with water simplifies process integration. Additionally, methanol has a high energy content and a high degree of reduction (γ_methanol_ = 6), which supports biosynthetic efficiency by providing reducing power for anabolic reactions and improving overall carbon and energy yields. A significant advantage is the existence of natural methylotrophic yeasts, which reduces the complexity required for metabolic engineering. Furthermore, compared to formate and CO₂, methanol has greater industrial readiness for renewable production and relatively low production costs (González-Garay et al. 2019).

However, several challenges remain. Firstly, regarding handling, methanol is both highly toxic and flammable, posing safety risks. This toxicity also affects bioprocess control, as excessive methanol concentrations can be detrimental to yeast cells, necessitating careful monitoring and regulation. Secondly, methanol’s toxicity presents obstacles when engineering yeasts, as it can induce metabolic stress, leading to reduced growth rates and product yields. Third, the native methanol metabolism in yeasts (e.g. via alcohol oxidase) is oxygen-dependent, complicating large-scale aerobic processes, particularly due to issues such as excess heat generation during oxygen supply.

**Figure 2.**
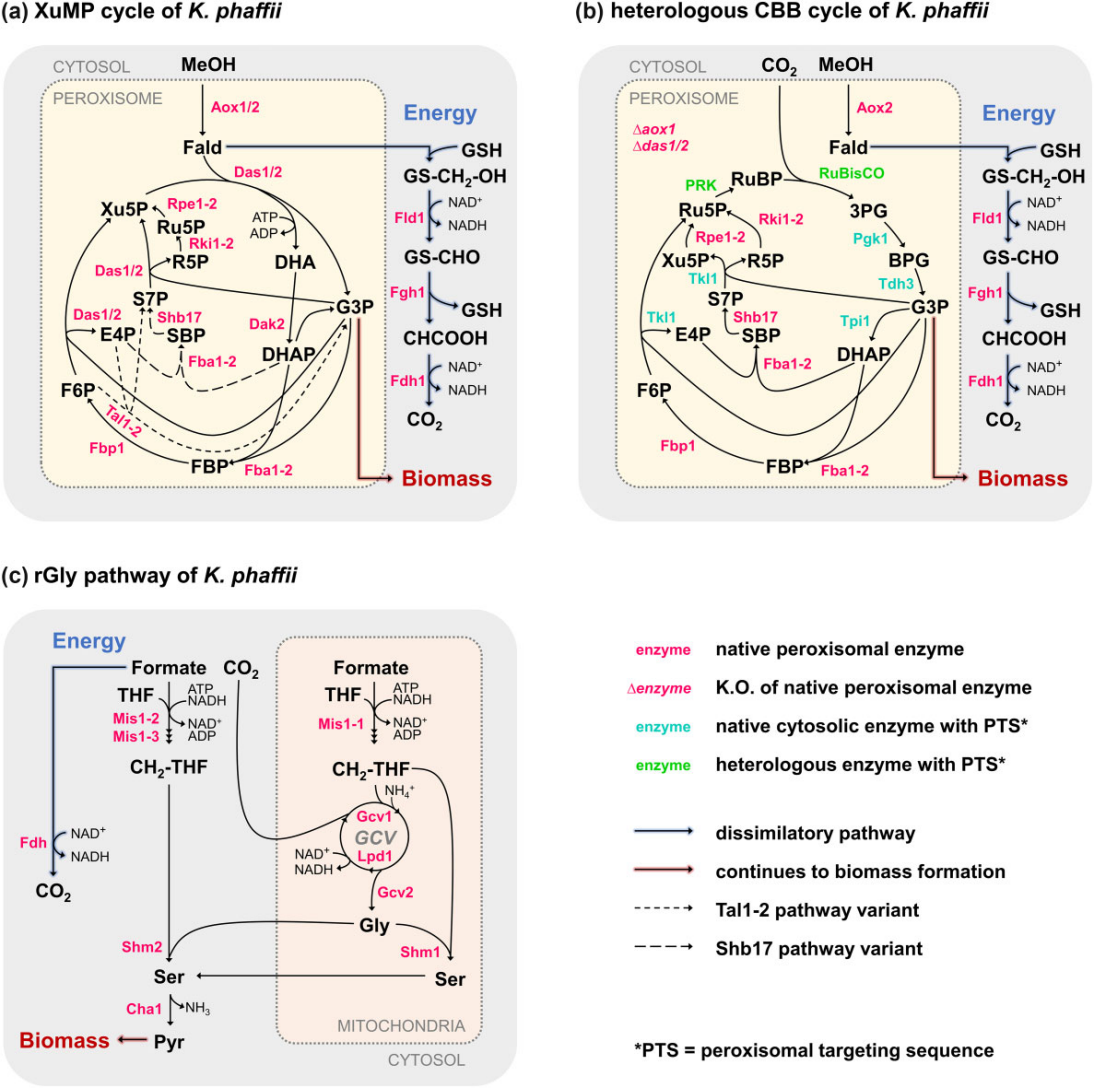
Metabolic pathways for C1 assimilation in yeasts. (a) Xylulose monophosphate (XuMP) cycle for methanol assimilation in methylotrophic yeasts. (b) Recombinant Calvin–Benson–Bassham (CBB) cycle, realized on the blueprint of the XuMP cycle in *K. phaffii*; native enzymes in pink, cytosolic enzymes targeted to peroxisome in cyan (PTS = peroxisomal targeting sequence), heterologous enzymes in green; *AOX1* and *DAS1/2* knockout (K.O.) (*Δaox1, Δdas1/2*). (c) Native reductive glycine pathway, identified in *K. phaffii*; GCV = glycine cleavage system. Enzyme abbreviations: Aox, alcohol oxidase; Cha1, catabolic l-serine (l-threonine) deaminase; Dak2, dihydroxyacetone kinase 2; Das, dihydroxyacetone synthase; Fld, formaldehyde dehydrogenase; Fdh1, formate dehydrogenase; Fba1-2, fructose 1,6-bisphosphate aldolase; Fbp1, fructose 1,6-bisphosphatase 1; Rki1-2, ribose 5-phosphate ketol-isomerase; RuBisCO, ribulose 1,5-bisphosphate carboxylase/oxygenase; Shb17, sedoheptulose 1,7-bisphosphatase; Fgh, S-formylglutathione hydrolase; Shm, S-adenosylmethionine hydrolase; Tkl1, transketolase 1; Tpi1, triose-phosphate isomerase 1; Lpd, dihydrolipoamide dehydrogenase; Mis, C1 tetrahydrofolate synthase; Pgk1, phosphoglycerate kinase 1; PRK, phosphoribulokinase; Tdh3, glyceraldehyde 3-phosphate dehydrogenase 3. Metabolite abbreviations: DHA, dihydroxyacetone; DHAP, dihydroxyacetone phosphate; E4P, erythrose 4-phosphate; Fald, formaldehyde; F6P, fructose 6-phosphate; FBP, fructose bisphosphate; GAP, glyceraldehyde 3-phosphate; GSH, glutathione; Gly, glycine; MeOH, methanol; R5P, ribose 5-phosphate; Ru5P, ribulose 5-phosphate; S1,7BP, sedoheptulose 1,7-bisphosphate; S7P, sedoheptulose 7-phosphate; Ser, serine; THF, tetrahydrofolate; Xu5P, xylulose 5-phosphate.

Methylotrophy in yeast is primarily driven by the XuMP pathway (Fig. 2a). Native methanol utilization via this pathway is restricted to the yeast genera *Komagataella, Ogataea*, and *Kuraishia*, and some species of *Pichia* and *Candida*. Additionally, in *K. phaffii* the linear rGly pathway has been identified (Mitic et al. 2023), enabling the conversion of formaldehyde into glycine (Fig. 2c). The XuMP cycle typically occurs in specialized compartments within the yeast, namely the peroxisomes, a strategy to mitigate the toxicity of the produced formaldehyde and hydrogen peroxide, and possibly to increase the local concentration of enzymes and intermediate metabolites. This localization to the peroxisome also needs to be considered during metabolic engineering when introducing synthetic pathways. Genes of interest must be targeted to the compartment using peroxisomal targeting sequences (PTS). With these technical specificities in mind, compartmentalization in yeast could, in fact, offer an opportunity to enhance the success of heterologous pathway introduction (Gassler et al. 2020). By sequestering toxic intermediates and separating overlapping reactions, this strategy provides metabolic flexibility and prevents interference with other essential cellular pathways that may run at different equilibria.

Comparing the XuMP cycle in yeast to other methanol utilization pathways found in nature highlights significant opportunities for metabolic engineering improvements. Specifically, the energy efficiency of the XuMP cycle is substantially lower than that of the RuMP cycle (esp. the variant using fructose bisphosphate aldolase and transaldolase; Trotsenko et al. 1996), one of the most energy-efficient pathways found in bacteria. Firstly, yeast utilizes oxygen as the electron acceptor instead of NAD(P)^+^ in the conversion of methanol to formaldehyde. Secondly, the RuMP cycle is three times more efficient in terms of ATP usage per glyceraldehyde 3-phosphate (GAP) molecule directed toward biomass formation, further impacting the overall efficiency of methanol metabolism.

Additionally, the dissimilatory branch of the XuMP cycle is associated with carbon loss in the form of CO₂, further reducing its overall efficiency. To address this challenge, Wang et al. (2024) attempted to improve the efficiency by inhibiting the dissimilatory branch through the knockout of formaldehyde dehydrogenase. To counterbalance the resulting imbalance in the NAD⁺/NADH ratio, they overexpressed isocitrate dehydrogenase, which helped to enhance flux through the tricarboxylic acid (TCA) cycle. Furthermore, they developed a dual-enzyme complex consisting of two key enzymes of the XuMP cycle, alcohol oxidase 1 (Aox1) and dihydroxyacetone synthase 1 (Das1), to reduce the accumulation of intracellular formaldehyde. In a prior study, the same group also demonstrated that the heterologous expression of methanol oxidase from *O. polymorpha* improved the methanol transformation rate in engineered *K. phaffii* (Wang et al. 2022). Notably, while the engineered strains showed a doubling time of 35 h, this rate remains relatively slow when compared to wild-type XuMP strains, suggesting further room for improvement.

An advantage of using and improving *K. phaffii* is that it is already well-established in various biotechnological processes, particularly for the production of heterologous proteins (Macauley‐Patrick et al. 2005, Gasser et al. 2013, Yang and Zhang 2018) and, more recently, for the synthesis of value-added chemicals (Zhu et al. 2019, Gao et al. 2021, Guo et al. 2021, Cai et al. 2022, Gao et al. 2024a, 2024b, Lu et al. 2024, Shen et al. 2024). Additionally, there is an existing, robust metabolic engineering toolbox available for *K. phaffii*, facilitating genetic modifications (Prielhofer et al. 2017, Peña et al. 2018, Gassler et al. 2019, Cai et al. 2021, Wu et al. 2024). However, it is often argued that large-scale industrial applications remain challenging due to the slow growth kinetics associated with the XuMP cycle (Guo et al. 2023).

Another native methylotroph and biotechnological yeast is *O. polymorpha*. This yeast was mainly subject to metabolic engineering efforts aiming to expand the product spectrum with methanol as a sole carbon source (Khongto 2010, Gao et al. 2022, Zhai et al. 2023, Li et al. 2024).

Efforts have been made to engineer synthetic methylotrophs from well-established biotechnological yeast species, with*Saccharomyces cerevisiae* being a prominent example. The first synthetic methylotrophic *S. cerevisiae* strain was developed by Dai et al. (2017) by introducing modules from *K. phaffii*’s XuMP cycle to enable the yeast to convert methanol directly to pyruvate. Espinosa et al. (2020) explored three different strategies to engineer a synthetic methylotrophic *S. cerevisiae*: (i) a heterologous XuMP cycle, (ii) a hybrid XuMP cycle featuring a bacterial methanol dehydrogenase (Mdh) instead of the yeast’s native *AOX1*, and (iii) a heterologous RuMP cycle. Their findings indicated that the RuMP cycle was the most efficient for methanol assimilation. To mitigate formaldehyde toxicity, the researchers enhanced *S. cerevisiae*’s native dissimilation pathway by overexpressing *SFA1*, which facilitated formaldehyde conversion to CO₂ while generating additional energy. Interestingly, they also discovered that *S. cerevisiae* possesses an inherent, albeit limited, capacity for methanol assimilation, which they successfully improved through adaptive laboratory evolution (ALE). Nevertheless, in liquid medium the addition of yeast extract is still necessary.

Zhan et al. (2023) engineered a synthetic methylotrophic *S. cerevisiae* strain using a modular circuit strategy, resulting in a strain capable of growth on methanol as a sole carbon source (2.3 cell doublings). More recently, Guo et al. published work employing the SCRaMbLE genome recombination technology, which allowed for genome rearrangement to exploit *S. cerevisiae*’s inherent methanol utilization capacity without relying on rational design. Subsequent ALE experiments resulted in a strain able to metabolize methanol via an Adh2-Sfa1-rGly (ASrG) pathway (Guo et al. 2024a).

Another yeast species engineered for methanol assimilation is *Yarrowia lipolytica*. Wang et al. (2021) introduced a chimeric methanol utilization pathway, combining elements of the RuMP and XuMP cycles. This enabled the yeast to assimilate methanol as a sole carbon source, achieving a methanol assimilation level of 1.1 g/l per 72 h. A different approach was taken by Zhang et al. (2023), where a heterologous XuMP cycle from *K. phaffii* was introduced alongside a xylulose utilization pathway, with further optimization achieved through compartmentalization in the peroxisome. The engineered methylotrophic *Y. lipolytica* strain successfully produced succinic acid in a proof-of-concept study (Zhang et al. 2023).

## Carbon dioxide

CO₂ is one of the major greenhouse gases contributing to the worsening climate crisis. The steady increase in CO₂ emissions since the Industrial Revolution reached over 425 ppm by February 2025, with an increasing trend highlighting the society’s role in driving global temperature rise (Lan et al. 2025). Total CO_2_ emissions including the land-use change are estimated to be higher than 40 billion tonnes in 2024 (Global Carbon Budget 2024). However, CO₂ could also become part of the solution to the problem it creates—if utilized as a carbon source, shifting its role from being a pollutant to a resource.

In nature, there are seven known CO_2_ fixation pathways: the CBB cycle, the rTCA, the rGly pathway, the oxygen-sensitive Wood–Ljungdahl pathway, the 3-hydroxypropionate (3-HP), the hydroxypropionate/4-hydroxybutyrate (HP/HB), and the dicarboxylate/4-hydroxybutyrate (DC/HB) cycles (Bassham and Calvin 1960, Evans et al. 1966, Ljungdhal 1986, Berg et al. 2007, Huber et al. 2008, Sánchez-Andrea et al. 2020).

Natural autotrophs are attractive hosts; however, their low carbon fixation efficiency and the limited availability of genome editing tools have shifted research focus toward more conventional hosts. To address these limitations, several yeast species, including *S. cerevisiae* (Guadalupe-Medina et al. 2013, Li et al. 2017, Xia et al. 2017, Papapetridis et al. 2018), *K. phaffii* (Gassler et al. 2020), and *Kluyveromyces marxianus* (Ha-Tran et al. 2021), have been engineered with the goal of developing strains capable of efficient CO₂ fixation.

A CBB cycle was successfully integrated into the yeast *K. phaffii*, enabling it to grow on CO₂ as its sole carbon source, while using methanol as the energy source (Gassler et al. 2020) (Fig. 2b). Subsequent ALE improved growth rates by increasing ATP availability and reducing the enzymatic activities of phosphoribulokinase (PRK) and nicotinic acid mononucleotide adenylyltransferase (*NMA1*) (Gassler et al. 2022). These findings emphasize the importance of carefully designing synthetic pathway integrations, as crosstalk between new and native pathways can significantly impact its efficiency. Notably, achieving higher efficiencies does not necessarily require high catalytic activity of the enzymes or overexpression of genes; instead, a balanced expression of genes and intracellular fluxes is essential. Additionally, this synthetic autotrophic yeast was engineered for the production of organic acids through the direct conversion of CO₂, demonstrating the potential of engineered yeast strains (Baumschabl et al. 2022). Although the production capacity of these strains is not yet competitive for economically viable processes, rapid advancements in synthetic biology could soon enable the development of yeast strains with faster and more efficient CO₂ fixation capabilities, contributing to future CO₂ mitigation efforts.

CO₂ has a degree of reduction of γ_CO2_ = 0, making it inherently challenging to utilize, as it requires additional energy sources to generate the necessary reducing power. Consequently, efforts to integrate synthetic CO₂ fixation pathways into yeasts require either an external energy source or the co-assimilation of CO₂ alongside other carbon and energy sources.

Accordingly, an alternative approach to enhance carbon efficiency is the utilization of CO₂ as a co-substrate through additional carbon fixation (Vásquez Castro et al. 2023). For instance, expression of ribulose 1,5-bisphosphate carboxylase/oxygenase (RuBisCO) and PRK, key enzymes of the CBB cycle, in *S. cerevisiae* has been shown to restore redox balance during ethanol production (Guadalupe-Medina et al. 2013). This modification decreased glycerol formation as a by-product, ultimately increasing ethanol yields. Similarly, xylose fermentation to ethanol by *S. cerevisiae* was improved with the co-expression of the RuBisCO-PRK module, where CO₂ facilitated the oxidation of excess NADH, contributing to redox balance restoration (Li et al. 2017, Xia et al. 2017). Comparable outcomes were observed in the thermophilic yeast *K. marxianus*, where the co-expression of Type I and II RuBisCOs enhanced ethanol production through higher rates of CO₂ fixation and improved redox balance (Ha-Tran et al. 2021). Furthermore, co-expression of molecular chaperones and the integration of multiple RuBisCO gene copies have been shown to further increase carbon fixation efficiency in these heterologous hosts (Guadalupe-Medina et al. 2013, Papapetridis et al. 2018, Gassler et al. 2020).

The exploitation of anaplerotic reactions offers an alternative strategy for the co-utilization of CO₂. In yeast, two key enzymes facilitate CO₂ fixation, leading to oxaloacetate production: phosphoenolpyruvate carboxylase (PEPC) and ATP-dependent pyruvate carboxylase (PC). Among yeasts, *S. cerevisiae* has been the predominant model for CO₂ fixation via anaplerotic pathways, largely through the overexpression of PC (Zelle et al. 2008, Xu et al. 2012, Kang et al. 2022). Research has focused on producing TCA cycle intermediates such as malate, fumarate, and succinate, as well as amino acids derived from these intermediates.

Advancements in synthetic biology and metabolic modelling tools have enabled the design of novel synthetic pathways with improved efficiencies. Among these, one of the most notable synthetic pathways is the oxygen-tolerant crotonyl-CoA/ethylmalonyl-CoA/hydroxybutyryl-CoA (CETCH) cycle, which employs the carboxylase crotonyl-CoA carboxylase/reductase (Schwander et al. 2016). More recently, additional synthetic pathways have been introduced, including the reductive glyoxylate and pyruvate synthesis cycle, the malyl-CoA-glycerate (MCG) pathway (Yu et al. 2018), and the POAP cycle (Xiao et al. 2022). The POAP cycle incorporates PC, oxaloacetate acetylhydrolase, acetate-CoA ligase, and pyruvate synthase; however, it is oxygen-sensitive and can only function under anaerobic conditions (Luo et al. 2022, Xiao et al. 2022). Despite the promising potential of these synthetic pathways, most are cyclic and consist of many novel enzymes, whose interactions with the host’s native metabolism remain unknown. Although the functionality of these pathways has been successfully demonstrated *in vitro*, their *in vivo* implementation remains challenging due to their complexity and the extensive rewiring of central metabolic fluxes they require. These challenges, along with strategies to overcome them, are discussed in detail elsewhere (Bierbaumer et al. 2023).

## Formate

Formate has emerged as a promising substrate in the field of C1 source utilization and holds a great potential, primarily due to the high efficiency of formate production through the electrochemical reduction of CO₂ compared to methanol (Wu et al. 2022). Its liquid state and water miscibility further enhance its compatibility for biotechnological applications. However, several challenges remain to be solved. Firstly, despite advancements, large-scale production of formate from CO₂ is still difficult, and no commercial applications have been established (Sánchez et al. 2019, Ewis et al. 2023, Izadi et al. 2023). Secondly, formate has a lower degree of reduction (γ_formate_ = 2 vs. γ_methanol_ = 6) and is less energy dense compared to methanol. Thirdly, natural formatotrophs are very rare in nature. Although the KEGG database lists over 90 reactions involving formate as either a reactant or product, only a few pathways for formate assimilation with limited knowledge of the regulatory elements are known. Two natural assimilation pathways have been identified: the serine cycle and two variants of the rGly pathway (Fig. 2c) (a selenium-dependent glycine reductase route and a selenium-independent serine-pyruvate route). Additionally, formate can act as an intermediate in the reductive acetyl-CoA (Wood–Ljungdahl) pathway or be directly utilized. Notably, formate assimilation is predominantly observed in prokaryotes (Crowther et al. 2008, Chistoserdova et al. 2009, Sánchez-Andrea et al. 2020, Song et al. 2020, Poehlein et al. 2024), though recent studies have shown that yeasts such as *S. cerevisiae, K. phaffii*, and *Y. lipolytica* possess endogenous genes for formate assimilation (Fig. 2c), thereby identifying them as potential hosts for formate assimilation (Gonzalez De La Cruz et al. 2019, Chen et al. 2024, Mitic et al. 2023).

Formate also acts as an electron source for energy production through its oxidation to CO₂, catalysed by NAD⁺-dependent formate dehydrogenases (FDH). These enzymes are also present in methylotrophic yeasts, where they contribute to generation of reducing power. However, due to the lower energy density compared to methanol, energy harvesting from formate to support growth is more challenging and requires further optimization. One potential strategy is to introduce multiple copies of native or heterologous FDHs to enhance formate utilization. Additionally, employing NADP⁺-specific FDHs (Calzadiaz-Ramirez et al. 2020) could improve growth by increasing the availability of mitochondrial methylene-THF, replenishing NADPH pools, and thereby enhancing the efficiency of the rGly pathway, as methylene-THF synthesis is NADPH-dependent.

Due to the limited knowledge in metabolic engineering and genome editing tools of the natural formate fixing organisms, several bacterial or yeast hosts are being exploited for synthetic formatotrophy (Gonzalez De La Cruz et al. 2019, Bysani et al. 2024, Guo et al. 2024b). This review focuses on yeasts; for recent advancements in bacterial expression systems, please refer to Yishai et al. (2017), Bang and Lee (2018), Kim et al. (2020), Turlin et al. (2022), Bruinsma et al. (2023), Kim et al. (2023), Tian et al. (2023), and Wenk et al. (2025).

*Saccharomyces cerevisiae*, with its endogenous rGly pathway genes, has shown significant potential for formatotrophy. Early studies demonstrated net glycine production through the rGly pathway in yeast (Gonzalez De La Cruz et al. 2019). Further studies emphasized the critical role of balancing key metabolites, such as C1-tetrahydrofolate (THF), and addressing the bottleneck of reducing power generation (Bysani et al. 2024). In a recent study (Guo et al. 2024), *K. phaffii* and *S. cerevisiae* were engineered to include a synthetic C1-assimilation pathway (MFORG), enabling the mixotrophic utilization of methanol or formate coupled with CO₂ fixation via the rGly pathway. This study further emphasized that energy harvesting during formate oxidation is a significant challenge compared to methanol. Furthermore, while both yeasts demonstrated remarkable flexibility to engineer for C1-substrate utilization, *K. phaffii* exhibited superior performance when grown on methanol or formate substrates.

Indeed, it has been shown that *K. phaffii* harbours a native, active oxygen-tolerant rGly pathway, and it is able to grow on methanol, formate, and CO_2_ without needing the expression of heterologous genes, however very slowly (14 days doubling time) (Mitic et al. 2023). Further metabolic engineering is required to fully unlock the potential of this strain, aiming to develop an innovative C1-based production platform with improved product yields, enabling net CO_2_ fixation.

Lastly, in addition to the challenges associated with rewiring metabolism for efficient formate assimilation, several cultivation challenges arise from the use of formate as a substrate. Notably, a positive correlation has been observed between alkalinization and formate consumption during the formatotrophic growth of both native (Collas et al. 2023) and engineered (Kim et al. 2020, 2023) bacteria utilizing the rGly pathway. This phenomenon is attributed to the uptake of formate in its protonated form (formic acid) by the cells. A formate transporter has not been characterized in yeast yet. However, a BLAST analysis using bacterial formate channel genes might help finding homologous sequences in both *K. phaffii* and *S. cerevisiae*, suggesting potential targets for formate uptake in yeast for further investigation.

## Outlook

**Figure 3.**
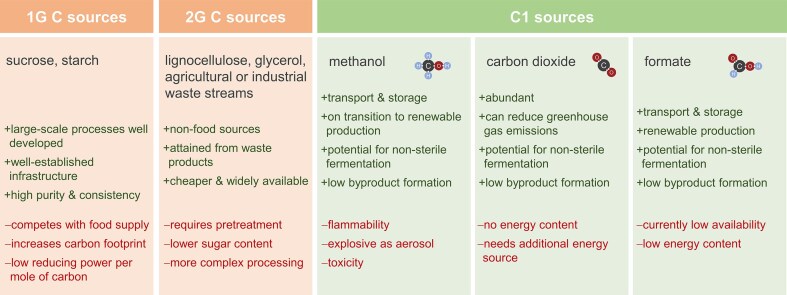
Advantages and disadvantages of different C1 carbon sources, in perspective with traditional substrates for industrial biotechnology.

Single carbon substrates provide valuable alternatives to agriculture-based carbon sources, which are mainly used in biotechnology today, as they can contribute to circular bioproduction without compromising land use for human nutrition. However, each of the three C1 sources discussed here has its advantages and disadvantages, and they depend on several factors: their chemical nature, availability, purity, and concentration, specifics of the assimilation pathways, as well as features of the products to be made from them (Fig. 3).

The following considerations may serve as a guideline to support decisions regarding which C1 substrate to consider for which type of process.

1. Assimilation pathway maturity: Methanol utilization is highly efficient as a native pathway that is well evolved over millions of years. CO_2_ utilization in yeast is fully synthetic, but it has been developed further quite far in the last 5 years. Formate assimilation in yeast has been demonstrated recently, a pathway is natively encoded in yeast, but it is probably not used for growth or metabolite production on formate in nature. It was demonstrated that this can be enabled but the level of maturity is lowest among the three substrates.
2. Features of the substrates: CO_2_ is a gaseous substrate with low water solubility, leading to limitations in process design and carbon transfer rates, while methanol and formate are liquid and highly water miscible. The liquid state of methanol and formate at ambient temperature and pressure favours their storage and transportation as well.
3. Nature of the product: The more reduced the product is, the more reduced a substrate should be. Comparing degrees of reduction is an easy help to make the right choice, whereby we should consider that methanol assimilation in yeast starts at the redox level of formaldehyde with γ = 4. As a rule, short-chain organic acids are usually rather oxidized (γ < 4), which favours the use of formate or CO_2_, while (poly)alcohols are more reduced (γ > 4) and are rather a point for methanol.
4. Energy balance of synthesis pathway: Assimilation of all three C1 substrates costs ATP, depending on their degrees of reduction (the more reduced, the less ATP is consumed). If a product pathway might release ATP further downstream, cellular energy could be rebalanced. However, it is more common that more ATP is consumed by the production pathway, so that generally the more energy-conserving assimilation routes are favoured.
5. Specific challenges of individual assimilation routes: Methanol assimilation in yeast begins with the wasteful oxidation to formaldehyde by alcohol oxidase, where the energy dissipates instead of being harvested as NADH. A possible solution would be to replace AOX by an alcohol dehydrogenase (Zavec et al. 2021). CO_2_ assimilation via the CBB cycle is very ATP costly. Possible solutions are other CO_2_ cycles, including synthetic cycles such as CETCH or other designs (Schwander et al. 2016, Dowaidar 2024). Formate is quite toxic to microbial cells, and its uptake shifts the pH towards alkaline due to the co-transport of protons. Possible solutions would mainly be found in bioprocess design with appropriate pH control and a controlled formate feed avoiding toxic concentrations.
6. Mixed feeds and co-assimilation: Several pathways co-assimilate CO_2_ with either formate or methanol. This has the advantage that different net degrees of reduction can be employed. Synthetic pathways integrated to the hosts may be designed to care for product-specific adjustment of mixing profiles to adapt for a redox-balanced metabolic process.

Considering different levels of maturity of C1-substrate-to-product processes, we observe a wide range of technology readiness levels between 2 and 7, with many attractive opportunities still requiring intense development. Given the broad interest and rapid developments in the field over the last few years, it can be anticipated that C1 substrates will be among the most important feedstocks for future circular bioproduction, and that the metabolic capabilities of yeasts make them important chassis organisms to reach this goal.
